# A disconnect between precursor frequency, expansion potential, and site-specific CD4^+^ T cell responses in aged mice

**DOI:** 10.1371/journal.pone.0198354

**Published:** 2018-06-04

**Authors:** Neha R. Deshpande, Jennifer L. Uhrlaub, Sing Sing Way, Janko Nikolich-Žugich, Michael S. Kuhns

**Affiliations:** 1 Department of Immunobiology, The University of Arizona College of Medicine, Tucson, AZ, United States of America; 2 The BIO-5 Institute, The University of Arizona College of Medicine, Tucson, AZ, United States of America; 3 The Arizona Center on Aging, The University of Arizona College of Medicine, Tucson, AZ, United States of America; 4 Division of Infectious Diseases, Cincinnati Children's Hospital Medical Center, Cincinnati, OH, United States of America; University of Iowa, UNITED STATES

## Abstract

T cell recognition of peptides presented within self-major histocompatibility complex (pMHC) molecules is essential for long-lived protective immunity. As mice age the number of naïve CD4^+^ and CD8^+^ T cells declines. However, unlike for CD8^+^ T cells, there are more naïve and memory phenotype CD4^+^ T cells that bind foreign pMHCII in old mice (18–22 months) than adults (12–15 weeks), suggesting increased promiscuity of pMHCII recognition with aging. Here we asked if CD4^+^ T cell responses to immunization or infection increase with aging since the magnitude of a CD4^+^ T cell response to a foreign pMHCII is proportional to the size of the precursor population in adult mice. We observed no difference in the number of pMHCII-specific CD4^+^ T cells in adult versus old mice for pooled secondary lymphoid organs after immunization, bacterial infection, or viral infection, but we did observe diminished numbers of pMHCII-specific CD4^+^ T cells in both the draining lymph node and brain of old mice after West Nile virus infection. These data indicate that an increased precursor frequency does not translate into more robust responses upon immunization or infection in old mice.

## Introduction

Aging is associated with reduced vaccine efficacy and increased susceptibility to infections [1–3]. These phenomena, which are collectively termed immune senescence, have been associated with a decline in the number or function of a variety of immune cells. For instance, age related defects in dendritic cell number, distribution, and function have been described [4, 5]. In addition, age-related decreases in the number of CD8^+^ T cells that bind foreign peptides embedded in class I major histocompatibility complex molecules (pMHCI) have been reported to correspond with reduced functional responsiveness to immunizations and infections [6–10]. How aging changes the CD4^+^ T cell compartment is less well studied. Specifically, intrinsic defects in T cell receptor signaling, IL-2 production, and memory cell generation have been described for CD4^+^ T cells from TCR transgenic mice [2, 11–13], but changes in polyclonal TCR repertoire, TCR affinity, and homeostasis of CD4^+^ T cells remain incompletely understood.

For polyclonal populations, it is established that the number of CD4^+^ T cells decreases in mice with aging [14, 15]. This is due to a loss of naïve cells (CD44^lo^) even though there is a marked increase in the representation of cells with a memory phenotype (CD44^hi^). Based on this decline in absolute numbers, a reasonable prediction would be that unprimed old mice (18–22 months) have a reduced number of cells that bind foreign pMHCII. But this is not the case. Instead, we have detected higher numbers of naïve and memory phenotype CD4^+^ T cells in old mice compared with adults (8–12 weeks) after enrichment with foreign pMHCII tetramers, indicating that the capacity of the CD4^+^ T cell repertoire to bind foreign pMHCII increases over the lifespan [15]. This fold-increase in CD4^+^ T cell populations that bind foreign pMHCII is related to their apparent tonic affinity for self-pMHCII (i.e. CD5 expression), homeostatic proliferation, and potential changes in thymic selection. To date, the consequences of these changes for CD4^+^ T cell responses to immunization or infection remains unexplored.

The lessons learned from studying CD4^+^ T cell responses in adult mice provide a clear framework from which to ask questions about whether and how aging changes CD4^+^ T cell responses upon immunization or infection. For example, it is well established that the pMHC-specific response magnitude of CD4^+^ T cells after immunization or infection directly relates to their precursor number [16, 17]. This has also been shown for monoclonal TCR transgenic (Tg) CD4^+^ T cell populations against a single pMHC in adoptive transfer experiments with adult mice [18]. In addition, a link between CD5 levels and responsiveness to foreign-pMHC has been proposed in some, but not all, systems [19–21]. If these rules govern CD4^+^ T cells throughout the lifespan then old mice would be expected to have larger responses than adult mice due to the increased pMHC-binding capacity and CD5 expression level of their CD4^+^ T cell repertoire.

However, the age-related changes that have been described to date for CD4^+^ T cells could easily be taken as evidence that the rules governing the CD4^+^ T cell compartment change over the lifespan [2, 11–13]. As a result, it is not unreasonable to expect that immunization or infection of old mice might elicit reduced CD4^+^ T cell responses. In addition, defects in the ability of adult CD4^+^ T cells to migrate to a draining lymph node in old mice have recently been reported, suggesting that age-related changes in the environment of old mice might lead to reduced CD4^+^ T cell responses upon immunization or infection [22]. Furthermore, the percentage of CD4^+^ T cells with a regulatory T cell (Treg) phenotype increases with aging and may be expected to impair responsiveness to immunizations or infection, although the existing data do not support such an impact [23]. Taken together, a reasonable prediction could be that CD4^+^ T cells should have a reduced responsiveness to immunization or infection in old mice compared with adults even if the mechanistic basis for such impairment may be multi-factorial and challenging to deconvolve.

The current study was conducted to increase our understanding of the impact of aging on CD4^+^ T cells. Specifically, we set out to enumerate pMHC-specific CD4^+^ T cells in adult and old mice after immunization or infection in order to evaluate how aging impacts their expansion. The many factors outlined above suggest that if the rules that govern CD4^+^ T cell responses in adult mice stay the same with aging, then an increased response would be expected. But, if they change, then a decreased or altered response might be predicted. We thus tested the null hypothesis that the rules stay the same over time such that CD4^+^ T cells have an increased response magnitude (i.e. increase in absolute numbers) after immunization or infection in old mice, when compared with adults, due to their increased capacity to bind foreign-pMHC as well as their increased tonic affinity for self-pMHC. Our results were inconsistent with this hypothesis, as we observed no difference in the absolute number of pMHC-specific CD4^+^ T cells between adult and old mice obtained from pooled secondary lymphoid organs after immunization or infection. This in turn reflected a reduced fold-expansion of pMHC-specific CD4^+^ T cells in old mice compared with adults. We did however observe a site-specific reduction in the numbers of pMHC-specific CD4^+^ T cells in the draining lymph nodes and brains of old mice, compared with adults, after infection with West Nile virus and these differences were not impacted by the depletion of Tregs. Collectively, the data suggest that the rules that govern CD4^+^ T cell response upon immunization or infection change over the lifespan. This is likely due to changes in the ratio of CD4^+^ T cells to APCs, intrinsic changes in the functionality of these populations and, perhaps most importantly, environmental changes that impact trafficking of cells to the right place at the right time to mount the appropriate response.

## Materials and methods

### Mice

Old (18–22 months) C57BL6/J, H-2^b^, mice were obtained from the National Institute of Aging breeding colony, Bethesda, MD. Adult (12–15 weeks) C57BL6/J mice were purchased from the Jackson Laboratory, Bar Harbor, ME. DEREG mice, a generous gift of Dr. Tim Sparwasser, were kept as hemizygous for the BAC Tg by crossing to C57BL6/J and aged at the University of Arizona [24]. All mice were maintained under specific pathogen-free conditions in the animal facility at The University of Arizona. Experiments were conducted under guidelines and approval of the Institutional Animal Care and Use Committee of The University of Arizona.

### Treg depletion

To establish Treg depletion, age-matched adult DEREG mice were injected i.p. with 1μg diptheria toxin (DT) or the inactive glu52 mutant as a control (Calbiochem) in 100μl of PBS for two successive days. The percent of FoxP3^+^CD4^+^ T cells in the spleen was assessed by flow cytometry using a Treg staining kit (eBiosciences) and determined to be 0.05% (not shown). This rebounded to 10% two days after the final DT injection. For WNV infection, littermate C57Bl/6 and DEREG adult and old mice were injected with 1μg DT in 100μl PBS on day -1 and day 0 of infection.

### Peptides and LPS

Synthetic peptides WNV Env641-655 (PVGRLVTVNPFVSVA) at >95% purity was purchased from 21st Century Biochemicals, Marlborough, MA. Purified LPS was purchased from Invivogen, San Diego, CA.

### Immunization

For peptide immunization, mice were immunized subcutaneously (s.c.) with 20μg peptide and 10μg LPS in PBS per injection on both sides of the base of the tail.

### Infections

*Lm*E641-OVA: The ActA-deficient *Lm*E641-OVA strain was produced using a similar approach as described previously by ligating the coding sequence for a chicken ovalbumin fragment fused to the WNV dominant epitope Env641-655 peptide into the Pst1 and Stu1 restriction sites of the pAM401-based *Lm*-expression construct [25]. Mice were injected intravenously with 10^7^ CFU *Lm*E641-OVA in 100μl PBS.

WNV: Mice were infected s.c. in the foot pad or between the shoulder blades with 1000 PFU of 385–99 WNV in 50μl of 1X PBS. Spleen, draining and non-draining popliteal lymph nodes were harvested on day 8-post WNV-infection. On day 10-post WNV-infection, spleen and brain (post-perfusion) were harvested.

### Tetramers and MHCII tetramer-based enrichment

Production of class II MHC tetramers and enrichment of CD4^+^ T cell populations with those reagents were performed as previously described [15].

### Brain processing

Mice were euthanized by isoflorane overdose to keep their circulatory system intact. Mice were perfused with 30mls of 1X PBS. Brain was collected in 2mls RPMI+5%FBS. Brain was disassociated over a 40μm strainer using the plunger of a 3ml syringe. The strainer was washed well with 15-25mls of RPMI+5%FBS. The suspension was centrifuged at 1650 RPM, 5 mins, 4°C and the pellet resuspended in 7mls of 30% Percol in a 15ml conical tube, centrifuged at 1650 RPM, 30 mins at room temperature or 4°C. Myelin and excess percol was aspirated off and the pellet was transferred to a new 15ml conical tube and washed with 10mls of RPMI+5%FBS followed by centrifugation at 2000 RPM, 5 mins, 4°C. The pellet was then transferred in a 96 well microtitre plate for surface staining [LIVE/DEAD yellow (Life Technologies, Carlsbad, CA), anti-CD3 (2c11, eBiosciences), anti-CD4 (RM4-5, eBiosciences), anti-CD8α (53–6.7, eBiosciences), E641:I-A^b^ tetramer].

### In-vivo proliferation assay

BrdU was delivered to mice through drinking water at the concentration of 1mg/ml + 1% glucose. Spleen and lymph nodes were harvested on day 6 post-immunization or infection. Following tetramer enrichment, cells were stained with cell surface antibodies [anti-CD3, anti-CD4, anti-CD19 (eBio (1D3), eBiosciences), anti-CD8α, anti-CD11c (eBiosciences), anti-F4/80 (BM8, Biolegend) and anti-CD5 (53–7.3, BD Pharmingen)] followed by intracellular anti-BrdU (BD Pharmingen) antibody according to the BrdU flow kit protocol (BD Biosciences).

### Statistical analysis

Mean fluorescent intensity of cell surface antibodies and intra-cellular antibodies were obtained from FlowJo software (Treestar, Ashland, OR). Comparisons were made using non-parameteric Mann-Whitey or ANOVA followed by Tukey’s posttest comparison. All statistical analysis was performed using GraphPad Prism software.

## Results

### Equivalent numbers of responding CD4^+^ T cells in adult and old mice after immunization

**Fig 1:**
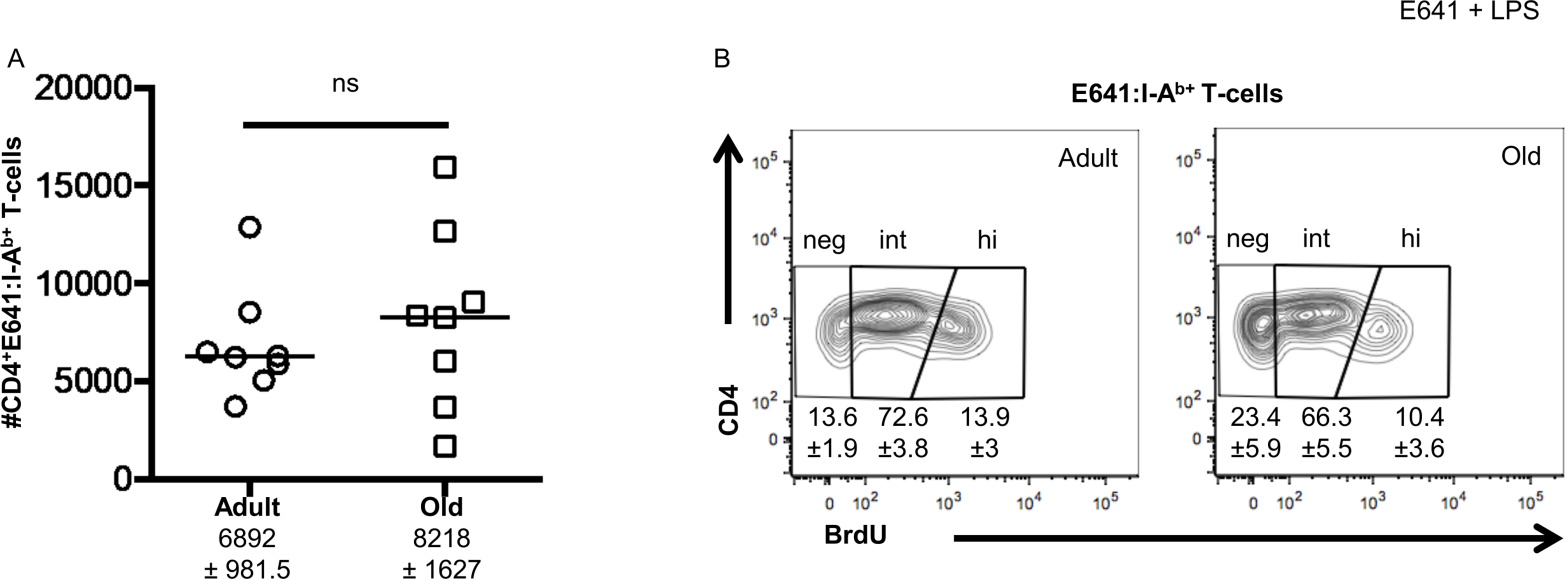
Disconnect between precursor number and response magnitude after immunization. Adult and old C57Bl/6 mice were immunized subcutaneously with 20μg peptide and 10μg LPS on both sides of the base of the tail and mice were kept on BrdU drinking water during the course of the challenge. Day 6 post immunization, pooled spleen and lymph node cells were incubated with E641:I-A^b^, OVA:I-A^b^ and MCC:I-E^k^ tetramers, enriched using anti-His magnetic beads, and enumerated by flow cytometry after antibody staining and gating on: lymphocytes via forward and side scatter; then CD3^+^ CD19^−^, CD11c^−^, F4/80^−^, CD8^−^ T-cells; then CD3^+^ CD4^+^ T-cells; and finally E641:I-Ab^+^, OVA:I-A^b−^, MCC:I-E^k−^ T-cells in the tetramer enrichment bound fraction from adult and old mice [15]. (A) Absolute number of E641:I-A^b+^, OVA:I-A^b−^, MCC:I-E^k−^ cells from adult and old mice after immunization with E641+LPS. Bars represent median values (ns p>0.05; Mann-Whitney). The average number per group (±SEM) is also shown below the respective label. (B) Concatenated contour plots showing BrdU incorporation by E641:I-Ab^+^, OVA:I-A^b−^, MCC:I-E^k−^ cells in adult and old mice (gated as above). E641:I-A^b+^ cells are divided into BrdU negative (neg), intermediate (int) and high (hi) subsets. Numbers indicate percent (±SEM) E641:I-A^b+^ CD4^+^ T cells in the respective gates. Shown are the aggregate results of two experiments with 4 mice/group.

We first asked whether having more CD4^+^ T cells that bind a particular foreign class II pMHC in old mice, compared with adults, translates to a larger responding population upon immunization. Adult and old mice were immunized with the immunodominant West Nile virus (WNV)-derived E641 peptide and lipopolysaccharide (LPS) was used as an adjuvant. The low antigen-complexity immunization was used to minimize the possibility of responses to additional foreign-pMHC, while E641:I-A^b^ tetramers were used to monitor pMHC-specific populations. This avoided the potential effects of self-peptides acting as coagonists and confusing the interpretation of a functional readout, as explained elsewhere [19]. On day 6 post-immunization, we enumerated equivalent numbers of E641-bound cells in pooled secondary lymphoid organs (SLOs) from both adult and old mice (**Fig 1A**). Given that contemporaneously analyzed unimmunized old mice had more total (CD44^lo^+CD44^hi^) E641-bound precursor cells (Median = 1092) than adult mice (Median = 600) [15], these data indicate that the fold expansion of E641-bound cells in old mice (based on median values) after LPS + E641 immunization (7.6x) was lower than in adults (10.5x). We also evaluated the proliferation of E641-bound cells after immunization. On average, old mice had a higher percentage of BrdU negative cells and a lower percentage of BrdU positive cells compared to adults, but these differences were not statistically significant in this sample size (**Fig 1B**). Altogether, the data indicate that a higher precursor number in unprimed old mice did not translate to a larger E641-specific response upon immunization in terms of absolute numbers of responders.

### Equivalent numbers of responding CD4^+^ T cells in adult and old mice after bacterial infection

We also evaluated CD4^+^ T cell responses to the gram-positive intracellular bacterium, *Listeria monocytogenes* (*Lm*), which has been used to study aging immunity in both mice and humans [26, 27]. In old mice, CD8^+^ T cells exhibit reduced response magnitude and effector functions in response to *Lm* [26]. We thus engineered an *Lm* expressing both the E641 peptide and OVA peptide (*Lm*E641-OVA) to examine if the increased number of E641- and OVA-bound cells in unprimed old mice results in: (i) an increased expansion of E641- or OVA- binding cells compared with adults; (ii) a comparable response in both adult and old populations; or, (iii) a reduced response.

**Fig 2:**
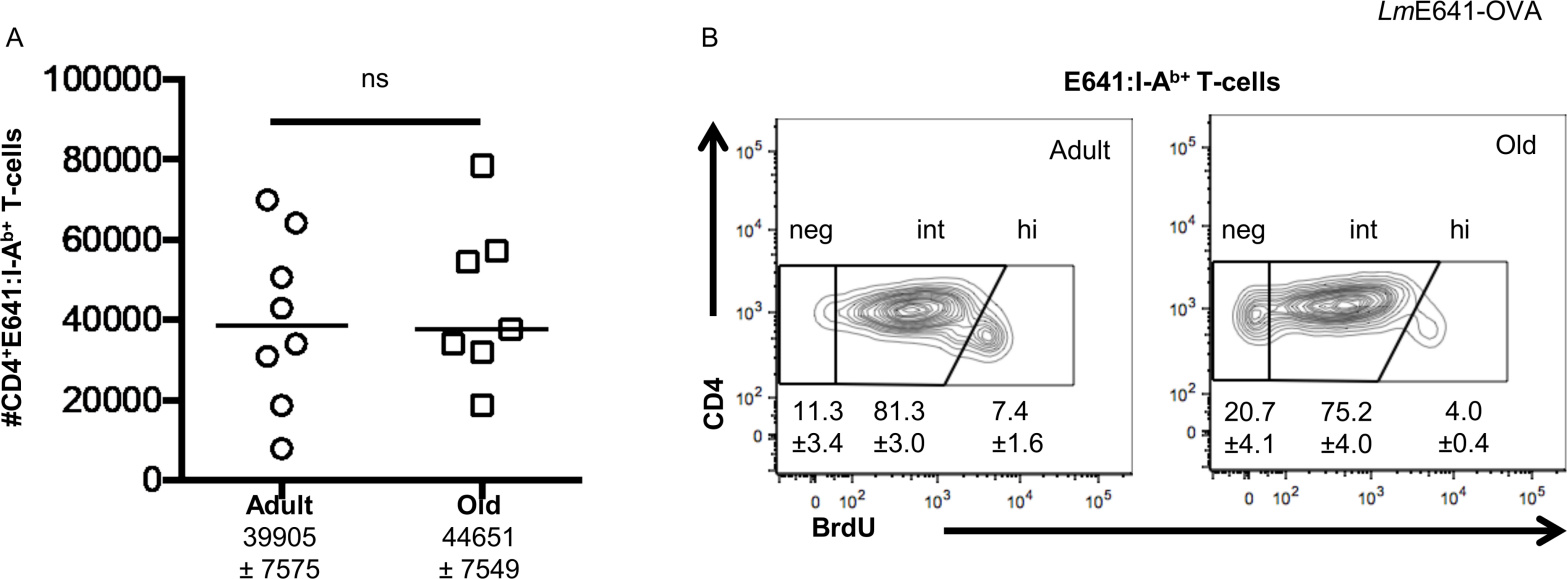
Larger precursor population does not equate to a larger response upon *Lm* infection. Adult and old C57Bl/6 mice were infected i.v. with 10^7^ CFU *Lm*E641-OVA in 100μl PBS. On day 6 post-infection, pooled spleen plus lymph node cells were incubated with E641:I-A^b^, OVA:I-A^b^ and MCC:I-E^k^ tetramers, enriched, and analyzed as in Fig 1. (A) Absolute number of responding E641:I-Ab^+^, OVA:I-A^b−^, MCC:I-E^k−^ CD4^+^ T-cells in adult and old mice (ns p>0.05; Mann-Whitney). The average number of cells (±SEM) is shown below the respective label. (B) Representative concatenated contour plot showing BrdU incorporation by E641:I-A^b^ only cells in adult and old mice. Numbers indicate percent (±SEM) E641:I-A^b+^ CD4^+^ T cells in the respective gates. Shown are the aggregate results of two experiments with 3–4 mice/group.

Here again, the number of E641-bound CD4^+^ T cells in pooled SLOs was equivalent in adult and old mice on day 6 post-*Lm*E641-OVA infection (**Fig 2A**). In addition, the old E641-bound population had, on average, a reduced percentage of BrdU positive cells and increased percentage of BrdU negative cells but the differences were not statistically significant in this sample size (**Fig 2B**). The OVA-bound cells did not expand (not shown). Since we previously showed that the OVA peptide is subdominant to the E641 peptide, this result is likely due to the immunodominance of E641 and other *Lm* peptides over OVA upon *Lm*E641-OVA infection [15].

### Site-specific differences in the number of WNV-specific CD4^+^ T cells in adult and old mice after WNV infection

We next evaluated the number of E641-bound CD4^+^ T cells in adult and old mice after subcutaneous WNV infection. These experiments were performed for multiple reasons. First, WNV lethality increases with age in both mice and humans, so we wanted to know if this was related to changes in the number of WNV-specific CD4^+^ T cells that expand upon infection [10, 28–30]. Also, recent evidence suggests that there are age-related defects in CD4^+^ T cell responses in the draining lymph node (dLN) of WNV-infected mice [22]. Thus, while we observed no overall differences in CD4^+^ T cell expansion to the immunodominant WNV epitope with LPS or Lm infection, here we wanted to analyze E641-bound cells in the non-draining LN (ndLN), dLN, and spleen to better determine if there might be local differences in expansion that might be missed upon pooling SLOs for tetramer analysis. Finally, Tregs are known to represent a higher frequency of the total CD4^+^ T cell population in old mice [23], so this numerical increase might have an inhibitory impact of WNV-specific CD4^+^ T cell expansion with aging; alternatively, Tregs have been shown to play a protective role in immunity to WNV infection, begging the question of whether that function changes with aging [31].

**Fig 3:**
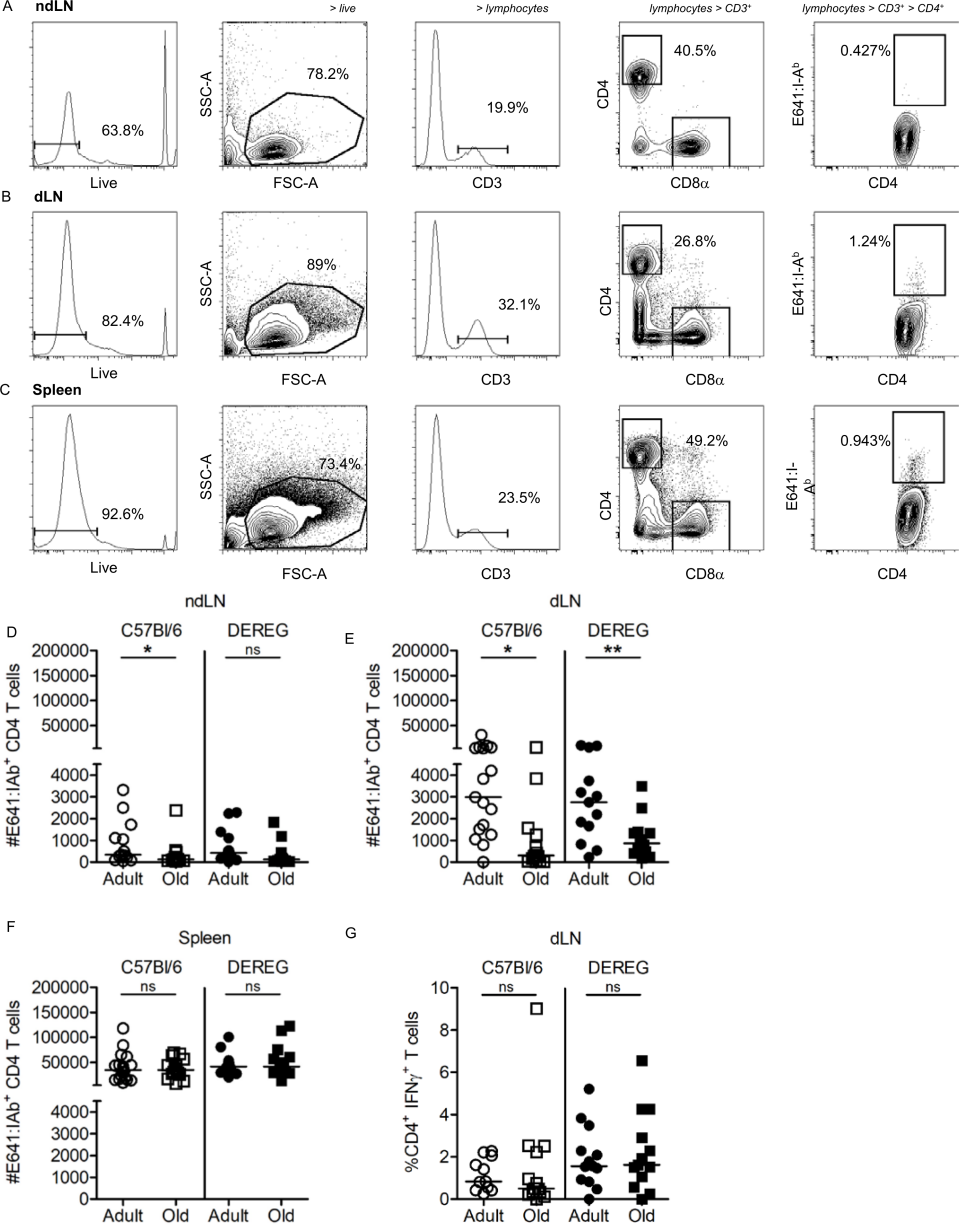
Fewer WNV-specific CD4^+^ T cells are present in the draining lymph node of old WNV-infected mice compared to adult. C57Bl/6 mice were infected with 1000 PFU 385–99 WNV, sc (foot pad). ndLN, dLN and the spleen were harvested on day 8 post-infection for lymphocyte analysis by flow cytometry (FCM). Representative gating strategy for CD4^+^ tetramer^+^ T cells via live, forward and side scatter, CD3^+^ T cells, CD8α^-^ CD4^+^ T cells and E641:I-A^b+^ CD4^+^ T cells harvested from (A) ndLN, (B) dLN, and (C) spleen are shown on day 8 post-WNV infection. Absolute numbers of E641:I-A^b+^ CD4^+^ T cells in adult and old C57BL/6 and DEREG mice enumerated for (D) ndLN, (E) dLN, and (F) spleen are shown as labeled. (G) Percent CD4^+^ INF-γ^+^ T cells in dLN of adult and old C57BL/6 and DEREG mice. Shown are the aggregate results of two experiments with (A-F) 6–9 mice/group and (G) 5–8 mice/group. Each dot represents a single mouse (*p<0.05; **p<0.01; Mann-Whitney).

To analyze E641-bound CD4^+^ T cells at different sites after WNV infection we deviated from tetramer enrichment analysis by enumerating WNV-specific CD4^+^ T cells in the ndLN, dLN, and spleen independently without following the enrichment protocol (**Fig 3A, 3B and 3C**). To couple these experiments with an analysis of Tregs, we infected adult and old control and DREG mice treated with DT to selective deplete Treg cells [24]. Here we used Treg-replete adult and old BAC Tg negative C57Bl/6 littermates treated with diptheria toxin (DT) as control mice to account for off target effects. This allowed us to compare the impact of Tregs across age groups.

For these experiments, we enumerated E641-bound CD4^+^ T cells on day 8 post-WNV infection (sc footpad). As expected, the numbers were low in the ndLN of both adult and old C57Bl/6 and DEREG mice (**Fig 3D**). In the dLNs, we detected more E641-bound CD4^+^ T cells in adult mice than old mice for both the C57Bl/6 and DEREG groups (**Fig 3E**). It is worth noting that the number of E641-bound CD4^+^ T cells trended higher in adult Treg-deficient mice than the Treg-replete adult mice (p = 0.07 Mann Whitney), but such a difference was less apparent in the old mice.

Despite the differences in the dLN between adult and old mice, the number of WNV-specific CD4^+^ T cells responding to disseminated virus in the spleen was equivalent in adult and old mice with or without Tregs (**Fig 3F**). As a result, aggregating the numbers of E641-bound cells from ndLN, dLN, and spleen of infected WNV-infected adult and old mice resulted in a statistically equivalent total SLO response magnitude between adult and old mice (not shown), as seen in the immunization and *Lm*E641-OVA infection. Altogether, these data suggest that the responsiveness of WNV-specific CD4^+^ T cells is impaired in the dLN of old mice but not in the spleen. This interpretation is consistent with a recent report of age-related defects in total CD4^+^ T cell responses to WNV in the dLN of old mice [22].

We also quantified the frequency of CD4^+^ T cells from the dLN making IFN-gamma after *in vitro* stimulation with the E641 peptide to assess the impact of aging on CD4^+^ T cell function. Here we saw no difference in the frequency of IFN-gamma-producing cells between C57Bl/6 or DEREG adult or old mice (**Fig 3G**). These data indicate that, although there was an age-related reduction in the absolute number of E641-bound CD4^+^ T cells in the dLN of old mice compared with adults (**Fig 3E**), the percentages of these cells that differentiated to a Th1 phenotype was not impacted.

**Fig 4:**
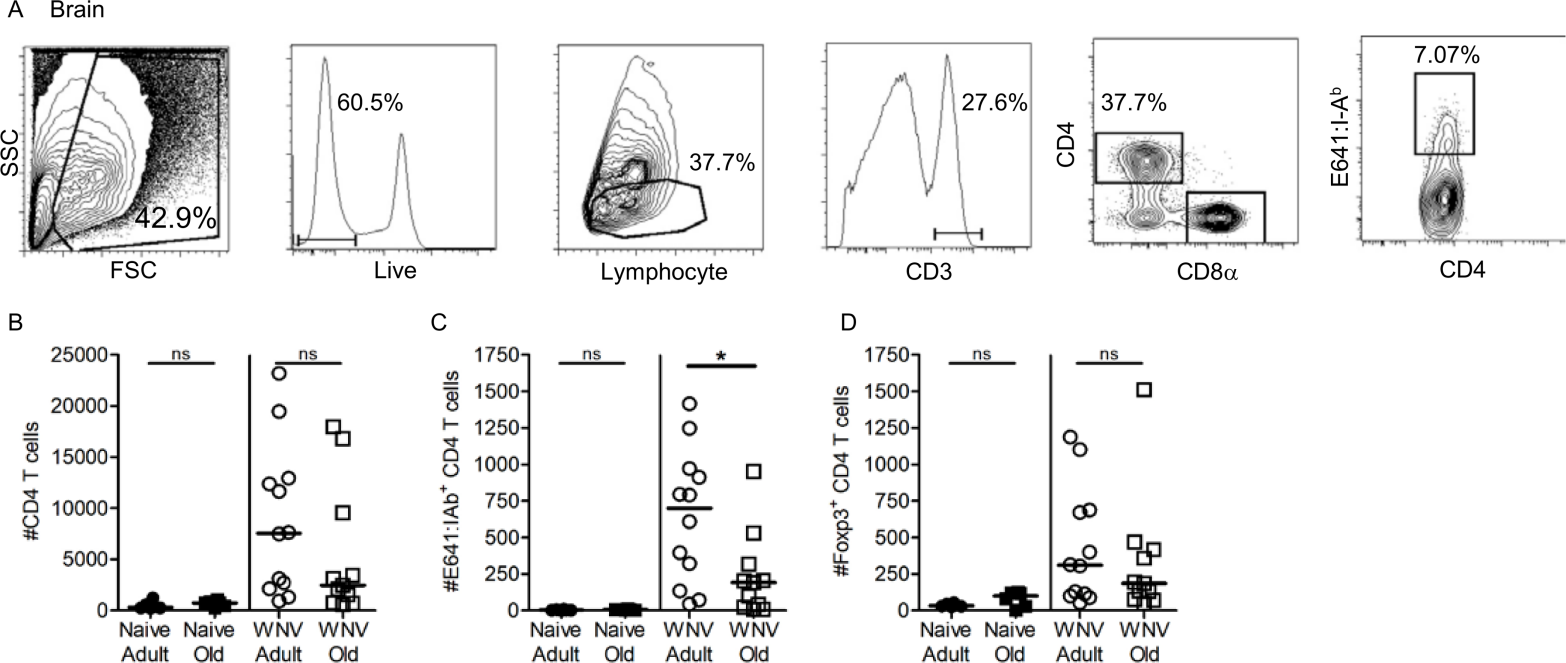
Reduced numbers of WNV-specific CD4^+^ T cells are present in the brain of old WNV infected mice compared to adult. Adult and old C57BL/6 mice were infected with 1000 PFU 385–99 WNV, sc (shoulder blades). Brain and spleens were harvested on day 10 post-infection and lymphocytes were analyzed by flow cytometry. (A) Representative gating strategy for CD4^+^ tetramer^+^ T cells via forward and side scatter, live, CD3^+^ T cells, CD8α^-^ CD4^+^ T cells and E641:I-A^b+^ CD4^+^ T cells harvested from brain on day 10 post-WNV infection. Absolute numbers of (B) CD3^+^ CD4^+^ T cells and (C) E641:I-A^b+^ CD4^+^ T cells and (D) Foxp3+ Tregs in the brain are shown as labeled. Results are representative of one of three experiments. Each dot represents a single mouse (*p<0.05; Mann-Whitney).

Finally, since the brain is the primary site of WNV infection and a lower percentage of CD4^+^ T cells accumulate at this site in infected old mice, we asked if the number of E641-bound cells in the brain on day 10 post-infection was different between adult and old mice (**Fig 4A**) [10, 32, 33]. This time point was chosen because it is the earliest day where virus can uniformly be detected but most old mice are still alive despite having two higher viral titres [10]. These experiments were not performed in the absence of Tregs since their loss accelerates lethality in adult mice [10, 31]. Consistent with prior work, we enumerated fewer CD4^+^ T cells in the brains of old mice compared with adults (**Fig 4B**) [28]. Importantly, there were significantly fewer E641-bound CD4^+^ T cells (**Fig 4C**) post-infection in the brain of old mice compared to the adults even though the number of E641-bound CD4^+^ T cells present in the spleen of these mice when comparable on day 10 (not shown). Finally, old mice do not have more Foxp3^+^ Tregs in the brain at this time point (Fig 4D). This suggests that local Treg suppression in the brain is neither limiting the expansion of WNV-specific T cells nor directly influencing mortality preferentially in old mice. Since old mice have more E641-bound CD4^+^ T cells than adults overall prior to infection [15], our combined results point towards an age-related defect in trafficking of the WNV specific CD4^+^ T cells to the draining lymph nodes, impaired immigration into the brain, impaired expansion upon secondary antigenic encounter in the brain, or some combination thereof. They do not suggest an increased Th1 response that might accelerate pathology. Such results are consistent with recent results documenting reduced trafficking of CD4^+^ T cells from old mice [22].

## Discussion

Decades worth of experiments with adult mouse and human T cells have provided important insights into the rules that govern the clonal representation and functionality of the adult T cell repertoire. Yet, little is known about the rules that govern T cell repertoires in aged individuals. With changing demographics worldwide, correcting this knowledge deficit is needed to inform the development of immunotherapeutics, including vaccines, which are tailored to the elderly.

Studies on T cells from aged mice and humans indicate that the T cell repertoire evolves with aging, resulting in changes in clonal representation and functional capacity [6–9, 15, 26, 34]. Immune senescence is thus not simply a relaxation of the rules that govern the system in its prime; rather, the rules change. For the CD8^+^ T cell compartment, there is both a reduced precursor number and responsiveness to foreign-pMHC. In contrast, the CD4^+^ T cell compartment acquires a broader recognition capacity for foreign-pMHCs with aging that is driven by the accumulation of cells with higher tonic affinity to self-pMHC [15]. The work presented here shows that an increased capacity of CD4^+^ T cells to bind foreign-pMHC does not translate into an increased response upon immunization or pathogenic infection in old mice. Instead, the recruitment and/or expansion of antigen-specific cells in the dLN and at the primary site of infection are compromised with age.

This age-related disconnect between precursor number and response magnitude is likely to be multi-factorial. Alterations in antigen uptake, pathogen sensing, and antigen presentation by CD8^+^α DCs change over the lifespan such that a numerical decline in APCs displaying cognate pMHC would result in a situation where only a fraction of pMHC-specific T cells may get recruited to a response due to competition for access to pMHC [5][35]. A finite and potentially limiting number of pMHC ligands or co-stimulatory molecules per APC could also result in the response of only those T cells in the pre-immune populations with the highest-affinity TCRs outcompeting others of the same specificity. In such situations, a larger pre-immune T cell population could produce a lower than expected response magnitude.

Age-related T cell intrinsic defects, or changes in immune cell trafficking, are also likely contributors. Intrinsic defects, including reduced TCR signaling and IL-2 production, are likely to underlie problems with the expansion of CD4+ T cells in old mice [2, 11–13]. Furthermore, a recent study reported age-related defects in trafficking of naïve CD4^+^ T cells to dLN [22]. The reduced number of E641-bound CD4^+^ T cells in both the dLN and brains of old WNV-infected mice are consistent with the idea that aging impacts the ability of CD4^+^ T cells to traffic to these critical sights and/or expand upon encounter of pMHC once there. Taken together such results point to age-related defects, at both the cellular and environmental level, that influence the recruitment, priming, and subsequent expansion of pMHC-specific responders upon WNV infection.

Finally, it is somewhat surprising that the absence of Tregs during WNV priming did not dramatically impact the number of WNV-specific CD4^+^ T cells in the dLN or spleen of either adult or old mice. The data clearly suggest that Tregs did not impact the number of E641-bound CD4^+^ T cells in the adult or old mice upon WNV infection. However, they do suggest the intriguing possibility that, in both adult and old mice, Tregs play an important role in regulating the differentiation of CD4^+^ T cells to a Th1 phenotype. This warrants further consideration in future studies with larger sample sizes.

The goal of studying age-related changes in the immune system is to acquire the knowledge needed to design therapeutic strategies aimed at providing long-lived immune protection. No obvious benefit was observed here for an increased capacity of the CD4^+^ T cell compartment to bind foreign-pMHC in old mice. Indeed, the overall response magnitudes were equivalent between adult and old mice, while responses to WNV in the dLN and brain were reduced in old mice. These data highlight that multi-factorial changes in the immune system impact CD4^+^ T cell responses over the lifespan and stress the need to decipher the ‘new’ rules that govern immunity in old individuals in order to better understand how to elicit the proper response in the proper place when needed.
